# Epigenetic Modifications and the Role of Medical Lasers in Enhancing Skin Regeneration

**DOI:** 10.1111/jocd.16780

**Published:** 2025-01-06

**Authors:** Diala Haykal, François Will, Hugues Cartier, Serge Dahan

**Affiliations:** ^1^ Centre Laser Palaiseau, Private Practice Palaiseau France; ^2^ Centre Laser Dermatologique Strasboug‐Rhin, Private Practice Strasbourg France; ^3^ Centre Médical D'arras, Private Practice Arras France; ^4^ Centre Esthétique et Laser de Toulouse, Private Practice Toulouse France

**Keywords:** collagen synthesis, DNA methylation, epigenetic modifications, medical lasers, skin regeneration

## 1 Introduction

As the demand for innovative and effective skin treatments grows, the field of dermatology continues to evolve, integrating cutting‐edge technologies with new biological insights. Among the most impactful advances in recent years are medical laser therapies, widely used to treat skin. While the immediate effects of laser treatments are well‐documented, research has begun to reveal that their benefits go deeper, potentially triggering long‐lasting changes at the molecular level. At the heart of this molecular transformation is epigenetics, focusing on changes in gene expression that do not alter the underlying DNA sequence. Unlike permanent genetic mutations, epigenetic changes can be reversible and are heavily influenced by environmental and external factors, including therapeutic interventions like laser treatments. Epigenetic mechanisms, such as DNA methylation, histone modification, and non‐coding RNA regulation, control many of the key biological processes involved in skin health, aging, and repair [1, 2]. Recent research suggests that medical lasers induce epigenetic modifications that enhance skin regeneration and repair, offering longer lasting results than previously understood [3]. This commentary aims to explore how lasers trigger long‐lasting regenerative effects through epigenetic mechanisms and how this knowledge could shape future advancements in dermatological care [4]. By understanding how lasers can tap into these epigenetic pathways, we open the door to more personalized and effective skin treatments that go beyond superficial improvements.

## 2 The Role of Epigenetics in Skin Health and Aging

The concept of epigenetics has revolutionized our understanding of how gene expression is modulated by external influences, including environmental exposure, stress, and injury. Unlike genetic mutations, epigenetic changes leave the DNA sequence intact but modify the way genes are expressed. These changes are reversible, offering a tantalizing avenue for therapeutic interventions in dermatology. In skin biology, epigenetic mechanisms play a critical role in processes like wound healing, inflammation, and aging [5]. Studies have shown that aging skin is associated with increased DNA methylation, which silences genes involved in collagen production and skin elasticity. Histone modifications, another key epigenetic mechanism, regulate the accessibility of chromatin, the structural framework of DNA, influencing gene transcription [3, 6]. In aging skin, histone acetylation and methylation patterns become dysregulated, contributing to reduced cellular repair and regeneration. Non‐coding RNAs, particularly microRNAs (miRNAs), have also emerged as important players in skin health. These small RNA molecules regulate gene expression at the post‐transcriptional level by binding to messenger RNAs (mRNAs) and preventing their translation into proteins. Specific miRNAs have been linked to the regulation of inflammation, collagen synthesis, and keratinocyte differentiation, all of which are critical for maintaining youthful, healthy skin [7, 8]. Laser treatments could modulate these epigenetic pathways by reprogramming molecular processes, resulting in more effective, longer lasting rejuvenation and improved overall skin health [9, 10].

## 3 Impact of Epigenetic Modifications on Clinical Outcomes

The epigenetic changes triggered by laser treatments contribute to the long‐term clinical improvements observed in skin rejuvenation, pigmentation correction, and scar reduction. For instance, fractional lasers, which create microthermal zones of injury surrounded by untreated skin, have been shown to improve the appearance of acne scars by promoting collagen production and tissue remodeling [11, 12, 13]. It is hypothesized that the regenerative effects of fractional lasers are partially due to epigenetic modifications, such as changes in histone acetylation that enhance the expression of collagen‐related genes [14]. In treating pigmentation disorders like melasma, which is characterized by hyperpigmentation due to the overproduction of melanin, laser treatments targeting melanocytes can modulate epigenetic pathways [15]. By influencing DNA methylation and histone modifications, lasers reduce the expression of genes involved in melanogenesis, leading to a more balanced and long‐lasting reduction in pigmentation. Laser‐induced epigenetic changes also offer promising results in the treatment of aging skin. Considering that in aging skin, there is a considerable dysregulation of the expression of collagen‐related genes and inflammation, ablative lasers stimulate a wound‐healing response that resets this dysregulation [12, 14]. Epigenetic modifications induced by the laser treatment can restore a more youthful gene expression profile, increasing collagen production and reducing the appearance of wrinkles.

## 4 Mechanisms of Laser‐Induced Epigenetic Modifications

Medical lasers are widely used in dermatology for a range of indications, from scar treatment to wrinkle reduction and skin resurfacing. Lasers work by delivering controlled energy to specific layers of the skin, causing microthermal damage that stimulates the body's natural repair processes [16]. The most commonly used lasers in skin rejuvenation include ablative (e.g., CO_2_ and Er:YAG lasers) and non‐ablative lasers, each varying in the depth and type of tissue injury they produce. What makes laser treatments particularly interesting from an epigenetic perspective is their ability to induce cellular stress [17]. This stress activates a cascade of repair processes in the skin, some of which are regulated by epigenetic modifications [18]. For example, the microdamage caused by laser treatment stimulates the inflammatory response, which in turn influences the expression of genes associated with wound healing, collagen production, and extracellular matrix remodeling. One of the primary epigenetic mechanisms influenced by lasers is DNA methylation, which involves the addition of a methyl group to the DNA molecule, typically at cytosine‐phosphate‐guanine (CpG) islands [3]. Laser‐induced inflammation leads to changes in DNA methylation patterns, silencing genes involved in the skin's aging process and promoting the expression of genes that regulate tissue repair. Histone modifications, particularly acetylation and methylation, are also affected by laser treatments. These modifications determine how tightly DNA is wound around histones, which in turn regulates gene accessibility [8, 13]. Studies have shown that histone acetylation is associated with active gene transcription, including genes that promote collagen synthesis and cell proliferation [19]. By inducing controlled injury, lasers would activate histone acetyltransferases, enzymes that add acetyl groups to histones, thus facilitating the expression of genes involved in skin repair. Additionally, laser treatments can impact the expression of non‐coding RNAs, particularly miRNAs. Research has demonstrated that certain miRNAs are upregulated following laser treatments, where they play a role in reducing inflammation and fibrosis while promoting epidermal regeneration [3].

The molecular changes induced by lasers, particularly in collagen synthesis, elastin production, and gene expression modulation, directly intersect with epigenetic processes. Lasers not only affect the physical structure of the skin but also influence gene activity by inducing epigenetic modifications that enhance cellular repair mechanisms and skin regeneration. For example, the activation of fibroblasts and keratinocytes leads to changes in the expression of key genes involved in collagen synthesis and extracellular matrix remodeling, closely tied to epigenetic processes such as DNA methylation and histone modifications. The reduction in matrix metalloproteinases and heat shock proteins after laser treatment indicates a shift in the cellular stress response, potentially through epigenetic pathways that regulate protein expression during skin repair [20]. This highlights how lasers can “reprogram” the skin's cellular environment for long‐term regenerative outcomes, offering new insights into how medical lasers can enhance skin health through both physical and epigenetic changes.

## 5 Lasers and Carcinogenesis Prevention

Recent studies have further explored the multifaceted role of laser treatments, not only in skin rejuvenation but also in preventing carcinogenesis through epigenetic mechanisms. Fractionated laser resurfacing (FLR) has demonstrated significant potential in preventing actinic neoplasia by re‐establishing IGF‐1 signaling in aged skin, thereby reducing UVB‐induced DNA damage and suppressing the formation of new actinic keratoses and non‐melanoma skin cancers [21]. Similarly, no ablative fractional lasers have shown promising prophylactic effects in patients with a history of keratinocyte carcinoma, where fibroblast activation and increased IGF‐1 expression contribute to a reduced recurrence rate [21, 22, 23]. In a pivotal randomized controlled trial by Spandau et al. 48 individuals aged 60 years and older with considerable actinic damage were treated with FLR. The study revealed that a single FLR treatment was durable in restoring appropriate UVB response in geriatric skin for at least 2 years, providing long‐term protection. Notably, FLR resulted in a sustained reduction in the number of actinic keratosis and a dramatic decrease in non‐melanoma skin cancers (NMSCs) on the treated arm (2 NMSCs) compared to the untreated arm (24 NMSCs) after 36 months [23]. This evidence suggests that laser therapies would act through epigenetic pathways, enhancing fibroblast function and keratinocyte repair while influencing collagen production and reducing matrix metalloproteinase activity [20]. The study by Pedersen et al. found that combining ablative fractional laser with the topical treatment vismodegib led to distinctive transcriptomic changes in early‐stage basal cell carcinomas in murine models. This combination treatment showed promise in modulating gene expression patterns associated with tumor growth inhibition, indicating that laser‐based interventions might enhance the efficacy of topical therapies in treating early carcinogenic lesion [24]. By modulating the skin's molecular environment, these treatments offer a protective and preventive role against carcinogenesis, highlighting the intersection of epigenetics, molecular biology, and advanced laser technologies in dermatological treatments.

## 6 Expanding the Scope: Beyond Skin Rejuvenation

In addition to skin rejuvenation, medical lasers play a crucial role in managing pigmentary and vascular skin disorders through epigenetic regulation, complementing their established role in enhancing skin structure and resilience. Lasers targeting pigmentary conditions like melasma influence melanin synthesis by altering gene expression through DNA methylation and histone modifications. By suppressing genes responsible for melanin overproduction, these treatments reduce hyperpigmentation and restore skin tone [15, 25]. Similarly, vascular lasers targeting conditions such as rosacea and hemangiomas modulate the expression of vascular endothelial growth factor (VEGF), promoting healthy angiogenesis while minimizing abnormal blood vessel formation. These effects demonstrate the versatile potential of lasers in regulating different skin pathologies via epigenetic mechanisms [26, 27, 28]. The therapeutic effects observed in treating pigmentary and vascular skin disorders align with the broader potential of lasers to modify cellular environments [29]. For example, DNA methylation can silence overactive pigment‐producing genes in melanocytes, while histone acetylation promotes the expression of genes involved in vascular stability and repair [30, 31]. Such interventions offer long‐lasting clinical benefits by reprogramming the skin's molecular landscape, highlighting the dual capability of lasers to address both aesthetic and pathological conditions [16].

## 7 Personalized Laser Treatments and Future Directions

As research in epigenetics continues to progress, the potential for personalized laser treatments based on individual epigenetic profiles is becoming increasingly feasible [32]. By analyzing a patient's unique epigenetic markers, such as DNA methylation patterns or miRNA expression, dermatologists can tailor laser therapies to optimize clinical outcomes and predict treatment responses. The development of laser technologies that can selectively modulate epigenetic pathways also holds significant promise for enhancing regenerative effects while minimizing adverse outcomes, such as post‐inflammatory hyperpigmentation. The integration of artificial intelligence (AI) and machine learning into this field is expected to further accelerate progress. AI's ability to process large data sets of genetic and epigenetic information will enable the identification of complex patterns and correlations, facilitating the development of predictive models for patient‐specific laser treatment responses [33]. This integration of precision dermatology and AI‐based analytics is poised to redefine clinical practice, offering unparalleled customization in laser‐based interventions [34].

## 8 Conclusion

The intersection of epigenetics and medical lasers presents a new frontier in dermatology, with the potential to significantly enhance clinical outcomes in skin regeneration, aging, and pigmentation disorders. By influencing gene expression through controlled cellular stress, lasers can tap into the skin's innate regenerative processes, offering long‐lasting improvements in skin health and appearance. As research into the epigenetic effects of laser treatments continues, the future holds more personalized, precise, and effective therapies that leverage the body's own epigenetic machinery to achieve optimal results. Incorporating an epigenetic perspective into laser therapy not only deepens our understanding of how these treatments work at a molecular level but also paves the way for innovations in skincare tailored to each individual's genetic and epigenetic profiles. This convergence of technology and biology has the potential to redefine dermatological care in the years to come.

## Author Contributions

D.H. contributed to the conception, drafting, and writing of the manuscript. F.W., H.C., and S.D. reviewed the manuscript. All authors have read and approved the final manuscript.

## Ethics Statement

The authors have nothing to report.

## Conflicts of Interest

The authors declare no conflicts of interest.

## Data Availability

The data that support the findings of this study are available on references' part.
